# *β*2 Integrin Signaling Cascade in Neutrophils: More Than a Single Function

**DOI:** 10.3389/fimmu.2020.619925

**Published:** 2021-02-18

**Authors:** Panagiota Bouti, Steven D. S. Webbers, Susanna C. Fagerholm, Ronen Alon, Markus Moser, Hanke L. Matlung, Taco W. Kuijpers

**Affiliations:** ^1^ Sanquin Research and Landsteiner Laboratory, Department of Blood Cell Research, Amsterdam University Medical Center, University of Amsterdam, Amsterdam, Netherlands; ^2^ Department of Pediatric Immunology, Rheumatology and Infectious Disease, Amsterdam University Medical Center (AUMC), Emma Children’s Hospital, University of Amsterdam, Amsterdam, Netherlands; ^3^ Research Program of Molecular and Integrative Biosciences, Faculty of Biological and Environmental Sciences, University of Helsinki, Helsinki, Finland; ^4^ Department of Immunology, The Weizmann Institute of Science, Rehovot, Israel; ^5^ Institute of Experimental Hematology, School of Medicine, Technical University of Munich, Munich, Germany

**Keywords:** CD11b/CD18 integrin, *β*2 integrin signaling, neutrophils, neutrophil function, therapeutic targets

## Abstract

Neutrophils are the most prevalent leukocytes in the human body. They have a pivotal role in the innate immune response against invading bacterial and fungal pathogens, while recent emerging evidence also demonstrates their role in cancer progression and anti-tumor responses. The efficient execution of many neutrophil effector responses requires the presence of *β*2 integrins, in particular CD11a/CD18 or CD11b/CD18 heterodimers. Although extensively studied at the molecular level, the exact signaling cascades downstream of *β*2 integrins still remain to be fully elucidated. In this review, we focus mainly on inside-out and outside-in signaling of these two *β*2 integrin members expressed on neutrophils and describe differences between various neutrophil stimuli with respect to integrin activation, integrin ligand binding, and the pertinent differences between mouse and human studies. Last, we discuss how integrin signaling studies could be used to explore the therapeutic potential of targeting *β*2 integrins and the intracellular signaling cascade in neutrophils in several, among other, inflammatory conditions in which neutrophil activity should be dampened to mitigate disease.

## Introduction

### Neutrophils

Human neutrophils or polymorphonuclear cells (PMNs) form the frontline of the immune response against bacteria and fungi. Despite their fundamental role in inflammation, recent evidence gives prominence to the controversial role of neutrophils in cancer (1, 2). Whereas recent data using deuterium labeling *in vivo* suggest that human neutrophils can survive up to 5.4 days in the circulation (3), general belief based on traditional studies still is that the high number of neutrophils emerging from the bone marrow on a daily basis (10^11^ per day) is balanced with their short half-life in the circulation (up to about 12h) (4). In contrast, the abundancy of neutrophil population in humans, representing up to 70% of circulating leukocytes, is not reflected on mice in which the percentage of neutrophils varies from 10 to 25% of white blood cells (5, 6).

Apart from the daily extravasation to offer that first natural line of defense at the tissue level, neutrophils are able to directionally migrate at larger numbers out of the bloodstream at the site of infection and either clear the pathogen or induce the recruitment of adaptive immune cells by chemokine/cytokine production or antigen presentation (7–9). Neutrophils exit the circulation *via* trans-endothelial migration, which takes place in sequential steps (tethering, rolling, crawling, cell arrest/firm adhesion/transmigration); all have been thoroughly described (10, 11). At the site of infection, neutrophils exert their killing properties to eliminate the pathogen extracellularly or upon phagocytosis. Neutrophils have the capacity to kill the invaded microorganism by secreting their toxic granular content (degranulation), producing reactive oxygen species (ROS), or releasing their DNA and subsequently forming neutrophil extracellular traps (NETs) in a process of cell death known as NETosis (6, 12). These processes of neutrophil extravasation, migration, and neutrophil effector functions against infectious agents rely to a very large extent on integrins.

### Integrins: Expression, Structure and Activation

Integrins are a family of ubiquitously expressed, transmembrane receptors. They anchor cells within their ambient extracellular matrix (ECM) and bind to counter-receptors expressed on other cells (13). Integrins are expressed on the cell surface as heterodimers of non-covalently associated *α* and *β* subunits (14, 15). So far, 24 different *αβ* heterodimers have been described in mammals, which exhibit specific ligand binding properties. The extracellular part of the *α* subunits defines ligand specificity. It consists of a seven bladed *β*-propeller module, followed by the thigh and two calf-domains (16–18). The *β* subunits consist of an N-terminal *β*A or *β*-I like domain, a plexin–semaphorin–integrin (PSI) domain, and *α* hybrid domain, followed by four cysteine-rich epidermal growth factor (EGF) repeats (19). While for most integrins the *β*-propeller from the *α* subunit and the N-terminal *β*A domain from the *β* subunit form the ligand binding head, a subset of vertebrate integrin *α* subunits, among them are the *β*2 integrin family, the collagen, and some laminin-binding integrins, have an additional *α*-I domain inserted into a loop in the *β*-propeller domain, which employ ligand binding (20). A hallmark of integrins is their ability to reversibly switch between different ectodomain conformations with distinct affinities for the ligand. It is meanwhile well accepted that integrins can adopt at least three conformations: a low affinity bent conformation, an extended-closed conformation with intermediate affinity, and an extended-open conformation with high ligand affinity (21–23). Notably, the difference in affinity for the ligand between the low and high affinity states varies strongly between different integrins from several hundred to several thousand fold (24–26). The conformational changes are either induced and stabilized by the extracellular ligand (outside-in activation) or are triggered by tightly regulated intracellular signals (inside-out activation); the latter is particularly important for integrins expressed on blood cells and will be described in detail in the following sections.

Both integrin subunits have a single-pass transmembrane helical domain, which are non-covalently associated within the plasma membrane. The two transmembrane domains interact at two interfaces termed the “outer membrane clasp” and the “inner membrane clasp”, which are mediated by helical packing of the N-terminal part and hydrophobic interactions and a salt bridge at the C-terminal part of the transmembrane domains, respectively (27–29). The transmembrane domains are essential for integrin function as they propagate conformational changes across the plasma membrane in both directions. These changes occur upon binding of the ligand to the ectodomain or intracellular adapter proteins to the cytoplasmic domain, which cause the disruption of the transmembrane domain interactions and a separation of the transmembrane and intracellular domains.

The intracellular domains represent the control modules of integrins. These rather short peptides serve as docking stations for numerous intracellular proteins, which regulate the integrin’s activity state and execute their functions. The majority of studies were done on the *β* integrin tail, which has a length of only 30 to 70 amino acids with the exception of the much longer *β*4 integrin cytoplasmic domain. Most *β*-tails contain a membrane proximal NPxY/F and a membrane distal NxxY/F motif flanking a serine/threonine-rich motif (30). Many intracellular proteins interact with these motifs in a phosphorylation dependent or independent manner. The most prominent proteins that bind to these motifs are Talins and Kindlins, which are essential integrin activators and will be described in detail below (31, 32). The cytoplasmic tails of the different *α* chains show low homology but share a GFFXR sequence in the membrane-proximal region (33). Much less is known about protein interactors, yet the tails of these *α* chains are important in the regulation of proteins bound to their respective *β* subunits (34). One of the few proteins known to associate with integrin *α* chains, including CD11a, is SHARPIN, an integrin negative regulator (35). In addition, CD11a, CD11b, and CD11c cytoplasmic domains have all been reported to be phosphorylated on conserved serine residues, and these phosphorylation sites are important for integrin function (36, 37).

### Integrin Activation by Talin and Kindlin

Intracellular signals that eventually trigger changes in integrin affinity for the ligand, also named as integrin inside-out activation, culminate in the binding of Talin and Kindlin to the *β* integrin tail. While Talin was long thought to be the sole integrin activator, studies in cells and animal models revealed that it requires assistance by Kindlin. Moreover, beside their essential function in integrin activation, both proteins initiate the formation of adhesion complexes that link integrins with the actin cytoskeleton and form signaling hubs that modulate many cellular processes (integrin outside-in signaling) (30, 31, 38, 39).

Talins are large cytoplasmic proteins consisting of an N-terminal head domain, which comprises an atypical FERM domain, and a C-terminal rod domain composed of 13 helical bundles. Talin1 is ubiquitously expressed and the major isoform expressed in hematopoietic cells, while the closely related Talin2 isoform shows a more restricted expression pattern (40). Binding of the Talin head to the membrane proximal NPxY/F motif within the integrin tail destabilizes the transmembrane interactions between the *α* and *β* subunits thereby inducing conformational changes of the integrin’s ectodomain (41). Talin is a mechanically regulated protein and provides a direct link with the actin cytoskeleton (42). It contains two actin-binding sites and several binding sites for the actin binding protein Vinculin within its rod (43, 44). Tensile forces generated by the actin-myosin contractile apparatus are transmitted *via* Talin to the integrin and contribute to full integrin activation. Moreover, the mechanical forces result in stretching and partial unfolding of several bundles within the Talin rod thereby exposing cryptic Vinculin binding sites (45–48). Before Talin is capable to bind and activate integrins it also requires an activation process as intramolecular interactions between the Talin head and rod domains preclude integrin binding (49). Details in the intramolecular interactions were recently provided by the cryo-EM structure of Talin. These structural data also revealed that even in the autoinhibited state the N-terminal F0 and F1 domains are exposed allowing them to bind to activated, membrane-bound Rap1 (50). This recruitment pathway is further promoted by electrostatic interactions between multiple sites within the Talin head and the negatively charged membrane lipids, in particular PIP2 (51). Concurrently, charge repulsions between the plasma membrane and the Talin rod domain interfere with the Talin head–rod interaction and promote the release in autoinhibition (52). Interactions with other proteins such as RIAM, Vinculin, and KANK proteins may further contribute to Talin recruitment and Talin activation and are further discussed below (53–55).

Mammals have three *FERMT* genes encoding Kindlin protein, which are expressed in a tissue specific manner. While Kindlin-1 is primarily found in epithelial cells, Kindlin-2 is almost ubiquitously expressed, and Kindlin-3 expression is restricted to cells of hematopoietic origin (56). The Kindlin protein structure resembles that of the Talin head and formed by an atypical FERM domain consisting of an N-terminal F0 domain followed by the canonical FERM domains F1, F2, and F3. A striking difference to the Talin FERM domain is an inserted PH domain within the F2 domain of Kindlins (57). The PH domain and a long loop within the F1 domain make contacts with phospholipids of the plasma membrane (58, 59). However, the molecular signals that direct Kindlins to integrin adhesion sites are not known yet. The Kindlin F3 domain binds directly to the membrane distal NxxY/F and the more N-terminally located threonine rich motif of the *β* integrin cytoplasmic tail (60, 61). Mutations in these motifs abolished Kindlin–integrin interaction resulting in defective integrin activation and function. Whether Kindlins alone, like as it has been shown for Talin, can induce conformational changes of the integrin ectodomain is not known yet. It rather seems that Kindlins support Talin-mediated integrin activation by further destabilizing the interactions between the integrin *α* and *β* transmembrane domains, which lead to the fully activated, extended open conformation of integrins (62, 63). Kindlins may also promote integrin activation by clustering integrins and thereby increasing their ligand binding avidity rather than their affinity (64). This model is also supported by structural data, which showed that Kindlin-2 can dimerize (65). Whether dimerization is a general mechanism of all Kindlin members to cluster integrins remains to be shown. Interestingly, recent crystallization studies suggest the formation of trimeric or even higher Kindlin complexes, which are unable to bind integrins (66, 67). Future studies will show whether Kindlin dimer/multimerization does occur and have an impact on integrin activity regulation *in vivo* and how this is regulated by upstream signals. Irrespective of their crucial role in integrin activity regulation more recent studies began to unravel the important role of Kindlins in adhesion site assembly and stabilization. Meanwhile multiple Kindlin interactors such as Paxillin, Leupaxin, ILK, and actin have been identified, and differences in the interactome or affinities to these and other still unknown interactors may explain the specific features of the individual kindlin members (68–73).

### Leukocyte Integrins and the *β*2 Integrin Family

In leukocytes, *α*M*β*2 or MAC-1 (CD11b/CD18), CR3; *α*Lβ2 or LFA-1 (CD11a/CD18); *α*D*β*2 (CD11d/CD18); *α*X*β*2 (CD11c/CD18); *α*4*β*1 or VLA-4 (CD49d/CD29); *α*5*β*1 or VLA-5 (CD49e/CD29); *α*6*β*1 or VLA-6 (CD49f/CD29); *α*4*β*7 (CD49d/HML-1), and *α*E*β*7 (CD103/HML-1) have been described (30, 74). *β*2 integrins are only found on leukocytes. The four members of this family are expressed on different leukocyte subpopulations and fulfil different roles in immunity. CD11a/CD18 is expressed on all leukocytes, regulating cell–cell communication by binding to ICAM ligands; it has the most restricted ligand repertoire of all the *β*2 integrins and is structurally more distantly related to the other three. In contrast, only myeloid cells including neutrophils express CD11b/CD18 and CD11c/CD18 on most cells, while macrophages also have additional CD11d/CD18 proteins on their surface, most likely with similar ligand binding properties (75). The most prominent integrin on neutrophils is CD11b/CD18, which has a promiscuous binding capacity to more than 40 ligands, including ICAM, iC3b, fibrinogen, and other ECM proteins (18), indicating its pleiotropic effect on multiple cellular processes.

The complexity of integrin biology is reflected in its intricate mechanism of activation. Recent reviews have proposed how *β*2 integrins get activated (31, 76). In brief, integrins may exist in three conformational stages, bent-inactive, extended-inactive, and extended-active conformation. Each of them is related to their binding affinity status and the subsequent efficacy of ligand binding. In fact, a recent report on *β*1 integrin suggests that all of the different integrin conformations can be present on the cell surface in a dynamic equilibrium in order to initiate or preserve cell motility (77). The “switchblade” and the “deadbolt” models have been predicted both describing the chronological order of integrin changes in affinity and ligand binding. However, there is no clear evidence supporting one of these models to date (31).

Focusing on the *β*2 integrins in leukocytes and, more specifically in neutrophils, neutrophils require the presence of Kindlin-3 protein for all CD11/CD18 integrin-mediated ligand binding (31). The importance of *β*2 integrin and their protein regulators has been demonstrated by the identification of human leukocyte adhesion deficiency syndromes (LADs) (78). Loss of *β*2 integrin in humans due to mutations in the gene *ITGB2* encoding the *β*2 subunit causes LAD-I. In contrast, LAD-III (or LAD-I/variant) arises from mutations in *FERMT3* encoding the Kindlin-3 protein (79–81) in the presence of normal levels of leukocyte integrins but a lack of high-affinity binding activity upon cell activation. Both syndromes result in similar patient phenotypic characteristics, showing susceptibility to bacterial infections due to the lack of neutrophil migration to the site of infection, poor wound healing, and late detachment of the umbilical cord in most affected children (82). In addition, many LAD-patients suffer from increased IL-17-driven periodontal tissue inflammation, leading to periodontitis, at least in part due to reduced recruitment of neutrophils (83). In LAD-III, the Kindlin-3 mutations also result in a clinical bleeding tendency due to the lack of *β*3 integrin activity on platelets (84). The role of Talin-1 in initiating intermediate integrin conformation and both Talin-1 and Kindlin-3 in inducing the high affinity state of the CD11a/CD18 integrin was subsequently reported in a murine model (63), very similar to CD11b/CD18 integrin activation by Kindlin-3 (85, 86). After initial activation, integrins engage with their ligands and form nascent clusters. Again, several models of clustering induction and stabilization have been suggested for *β*1, *β*2 or *β*3 integrins, yet the precise mechanism remains to be elucidated (87–90).

Although Talin-1 and Kindlin-3 are the primary proteins associated with *β*2 integrins in case of both inside-out and outside-in integrin activation, *β*2 integrins can only function effectively after recruitment of several additional proteins to the *β*2 integrin cytoplasmic tail, thus regulating or stabilizing its high-affinity state. Although recent technology can identify proteins associating with integrins (91), the heterogeneity among the different *β* integrin subunits as well as their diverse function in different cell types averts a general statement about integrin signaling and function. Although the proteins that can directly associate with *β*2 integrins or regulate their affinity and function have been reviewed (92), their contribution to downstream signaling of *β*2 integrins in the context of neutrophils is limited (38, 93–97). Examples of proteins interacting directly with the cytoplasmic tail of the *β*2 subunit and having a known function in neutrophils include Cytohesin-1 (98, 99), SYK (100, 101), and Filamin A (97) as we will also discuss below.

## Inside-Out Signaling During Endothelial Transmigration

### Chemokine Mediated Signaling

For trans-endothelial migration to occur, leukocytes utilize CD11a/CD18 and CD11b/CD18 to adhere to the endothelial wall. As discussed, CD11a/CD18 and CD11b/CD18 are kept in an inactive state, and require activation through recruitment of Talin-1 and Kindlin-3. Activation of these *β*2 integrins increases their binding affinity, which allows adhesion of leukocytes to several substrates including endothelial membrane proteins ICAM-1, upregulated during inflammation, and ICAM-2. During selectin-mediated rolling and slow rolling, leukocytes engage with chemokines and cytokines presented on the vessel wall with several different receptors, of which most are G-protein coupled receptors (GPCRs), also known as seven transmembrane receptors. Engagement of chemokines, such as CXCL8 (better known as IL-8) and CXCL1 (also known as GRO-*α*, MGSA, or KC) with their respective receptors causes the activation of CD11a/CD18 and CD11b/CD18 through a common pathway which recruits Talin-1 and Kindlin-3 to the cytoplasmic tail of CD18. Note that Talin-1 can be recruited through both selectin and GPCR signaling.

**Figure 1:**
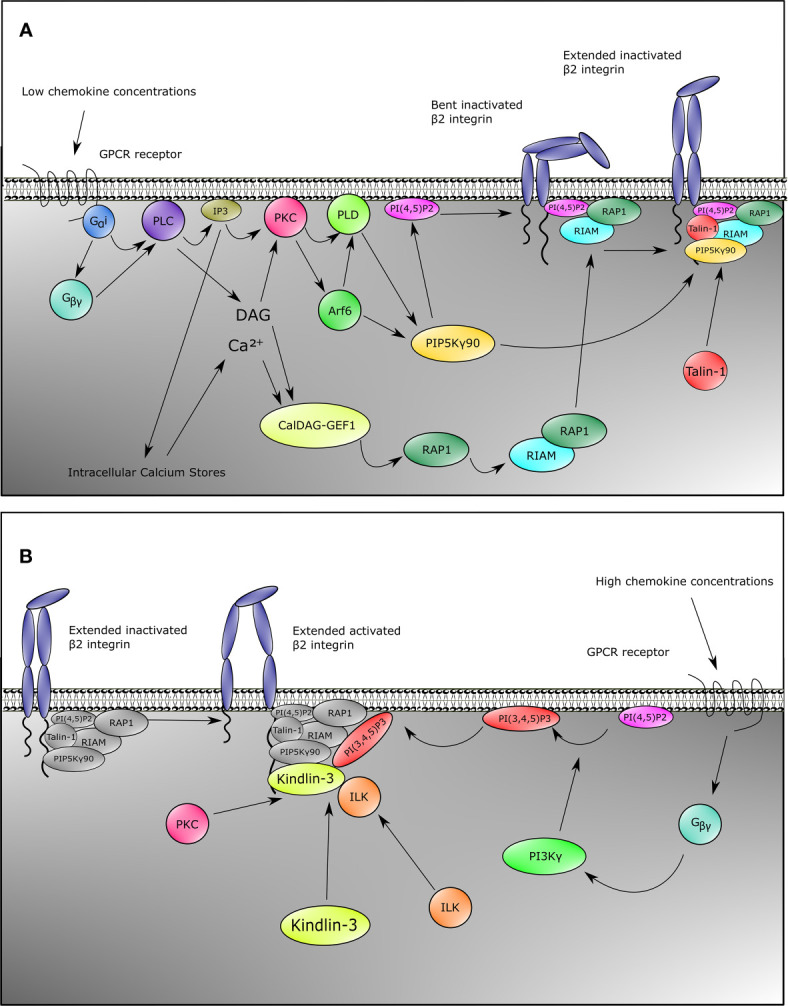
Inside-out signaling recruits Talin-1 and Kindlin-3 to *β*2 integrin. Upon stimulation of neutrophils with chemokines, both Talin-1 and Kindlin-3 become rapidly recruited to activate *β*2 integrins. This can be divided into two pathways for Talin-1 and Kindlin-3. For Talin-1 recruitment **(A)**, GPCRs engage with their ligands which causes the heterotrimeric G-proteins to split into G*_α_* and G*_βγ_*. This initiates the activation of PLC by G*_α_*_i_, and G*_βγ_*. Signaling through the second messengers’ calcium which is released from the endoplasmic reticulum by IP_3_ and diacylglycerol (DAG) is a critical element in many biological systems. Integration of calcium and DAG signals has been suggested to occur primarily through protein kinase C (PKC) family members, which bind both calcium and DAG. An alternative pathway involves CalDAG-GEF1, which has structural features (calcium-binding EF hands and DAG-binding C1 domains) and functions in calcium and DAG signal integration. This GEF activates RAP1, which then results into activation of RIAM and binding of RIAM to Talin-1. Together with catalyzation of PIP into PI(4,5)P_2_ by PIP5K_γ_90 through the PKC-PLD-Arf6 axis, these events recruit Talin-1 to *β*2 integrin, thus extending the integrin from the bent conformation. G*_βγ_* is also involved in Kindlin-3 recruitment **(B)**. In addition to activating PLC, G*_βγ_* also activates PI3K, which generates PI(3,4,5)P_3_. This allows recruitment of Kindlin-3 to *β*2 integrin. Binding of Kindlin-3 after Talin-1 binding activates extended *β*2 integrins. Note that the Kindlin-3 recruitment cascade might not be complete.

This common pathway through GPCRs starts with G*α*_i_ and the G*β*/*γ* subunits, which split after chemokine engagement with the receptor and activate Phospholipase C *β*2 (PLC*β*2) (**Figure 1A**) (102–104). The signaling then branches into two pathways, as has mostly been supported by gene knockout models in mice. The first is the activation of CalDAG-GEFI (RASGRP2) by PLC*β*2 and to a minor extent PLC*β*3 through the generation of DAG and a calcium influx by IP3 generation (105, 106), resulting in the activation of Rap1 (107). Activated Rap1 exposes a membrane targeting sequence, and upon interaction with the plasma membrane the N-terminus of Rap1 interacts with RIAM, which contains a Talin-binding sequence (108). A reduced fusion protein consisting of the Rap1 membrane targeting domain and the RIAM-Talin-binding sequence is sufficient to target Talin-1 to the plasma membrane, allowing engagement of Talin-1 with the NPXY motif in the cytoplasmic tail of CD18 (109) and resulting in *β*2 integrin activation (108). Interestingly, direct Rap1 interactions have also been described with the F0 and F1 domains of Talin-1, which in mice are pivotal for normal overall integrin activation, adhesion, and cell spreading, whereas the RIAM–Talin-1 interaction seems specific for *β*2 integrins in leukocytes (110–113).

The second pathway for *β*2 integrin activation downstream of PLC goes through Protein kinase C (PKC). PKC is activated by both DAG and IP3 generated by PLC. PKC then elicits its function by activation of Phospholipase D (PLD) and Arf6 (**Figure 1A**) (114, 115). Both Arf6 and PLD subsequently activate PIPKI*γ* (also known as PIP5K1C) which increases phosphatidylinositol 4,5 (PIP_2_) levels at the inner plasma membrane (104, 114–117), resulting in the recruitment and activation of Talin-1 *via* its PIP_2_ binding F2 domain and subsequent binding to the NPXY motif of the CD18 cytoplasmic tail (**Figure 1A**) (104, 109, 112, 118). Also taking into account a direct interaction between the C-terminal domain of PIP5K(*γ*90) and Talin-1, this collectively suggests a feedforward loop of both recruitment of Talin-1 to the *β*2 integrin tail and activation of Talin-1 through local increase in PIP_2_ levels close to the *β*2 integrin (118).

Although multiple studies have shown the importance of Kindlin-3 for *β*2 integrin activation (31, 63), the signaling pathway essential for activation of Kindlin-3 and recruitment to the CD18 cytoplasmic tail is as yet still less clear compared to what is known about Talin-1 activation (**Figure 1B**). Notwithstanding, Kindlin-3 recruitment to CD18 temporally precedes the high affinity conformation of *β*2 integrins (59). Additionally, structure function analyses have indicated that the pleckstrin homology domain of the F2 subdomain of the FERM domain is important for binding phosphatidylinositols at the plasma membrane (119, 120), and that part of this protein population has been suggested to reside in an auto-inhibitory homotrimer (67). Moreover, high chemokine concentrations induce neutrophil arrest, which is mediated through high-affinity *β*2 integrin by Kindlin-3 associating with the cytoplasmic tail of the *β*2 subunit and PIP_3_ increments upon strong activity of PI3K (**Figure 1B**) (104). Blocking PI3K has been suggested to decrease human neutrophil adhesion to fibrinogen, and adhesion to ICAM-1 is affected by specific blocking of PI3K with wortmannin (103, 121). ILK might also play a role in Kindlin-3 function in mice since genetic abblation resulted in reduced Kindlin-3 phosphorylation and a decrease in high-affinity CD11a/CD18 (122).

Despite these common pathways in *β*2 integrin activation, some chemokines trigger alternative pathways. For instance, in murine neutrophils, CXCL1 induces a strong calcium influx, which is an important signaling mediator (**Figure 1A**) for *β*2 integrin activation (123). This differential signaling effect is substantiated by the knockout of *TRPC6* encoding a plasma membrane calcium channel, resulting in lower calcium transient increases, thus leading to reduced Rap1 and *β*2 integrin activation by CXCL1 (124). Other chemokines remain to be tested for this pathway. Secondly, the actin-binding protein Hematopoietic cell‐specific LYN Substrate 1 (HS1) knockout mice have reduced Rac1 and subsequent Rap1 activation upon CXCL1 stimulation (125), which indicates another integrin activation pathway parallel to the common GPCR pathways (**Figure 1A**) in at least CXCL1-mediated integrin activation. Additionally, IL-8 also branches towards another pathway *via* PI3K, which activates the serine/threonine kinases RAF-1 and B-RAF, from which RAF-1 is expressed in human neutrophils. Both RAF-1 and B-RAF can subsequently activate the MEK/ERK/p38-MAPK pathways (126), and blocking MAPK indeed reduces neutrophil adhesion to fibrinogen, indicating a role for this pathway in CD11b/CD18 activation (103).

There are some noticeable differences in the activation of CD11a/CD18 and CD11b/CD18, which can solely be attributed to the presence of either CD11a or CD11b. For example, Cytohesin-1 is known to activate CD11a/CD18 and increases firm adhesion, spreading, and transendothelial migration for mouse neutrophils, whereas the opposite effect was observed for CD11b/CD18 (98, 99, 127–129). Additionally, CD11a Ser^1140^ and CD11b Ser^1126^ phosphorylation are critical events in the activation of the respective CD18 heterodimers for binding of ICAM-1, to a lesser extent ICAM-2, but not for the non-cellular ligand inactivated complement 3 fragment b (iC3b) in case of CD11b/CD18 (36, 130). Furthermore, CD11a/CD18 in humans is more rapidly activated than CD11b/CD18, but the activation is more transient, being deactivated after 1 min. In contrast, CD11b/CD18 seems to become fully activated after 1 min and remains activated for up to more than at least 5 min after stimulation with fMLP (131). This may reflect different roles of CD11a/CD18 and CD11b/CD18 in trans-endothelial migration, where CD11a/CD18 facilitates slow rolling and early adhesion, whereas CD11b/CD18 has a more prominent role during firm adhesion and subsequent chemotaxis. These findings are speculative and should be interpreted against background expression levels of these integrins, being much higher for CD11b/CD18 on human but not murine neutrophils. Selective genetic defects for either of the two integrins in gene knockout models of the respective *α*-chain have not been fully explored and cannot be supported by human ‘experiments of nature’ with genetic mutations in patients as the cause of disease.

### Selectin-Mediated Signaling

Aside from GPCRs, other mechanisms of inside-out signaling have been described. Selectin-mediated signaling has been thoroughly described in the past (132), and it is out of the scope of this review, yet we briefly discuss the concept in response to *β*2 integrin activation. During selectin-mediated rolling, the physical interactions between L-selectins on leukocytes with P- and E-selectins on inflamed endothelium induce recruitment of Talin-1 to the cytoplasmic tail of CD18 (133). Upon engagement of L-selectin with E-selectin, Src-family kinases (SFKs) are recruited and phosphorylate either FcR*γ* adapters or DAP12 (134) together with ERM proteins Moesin and Ezrin in an ITAM-motif based manner (135). This is followed by phosphorylation of Spleen tyrosine kinase (SYK) (136), which then recruits a complex of SLP76 together with ADAP (137). These two proteins subsequently activate Bruton tyrosine kinase (BTK) to phosphorylate p38-MAPK and also activate PLC*γ*2 (133) instead of PLC*β*2 (as known for chemokine stimulation). Downstream signaling through selectins involves the activation of Rap1a and PIP5K(*γ*90) (**Figure 1A**) (104), thus leading to the convergence of selectin-mediated signaling with the common GPCR pathway resulting in the recruitment of Talin-1 to CD11a/CD18 and CD11b/CD18 (110, 111, 138). To date, selectin-mediated CD11a/CD18 activation has only been reported for murine and not human neutrophils, which coincides with a much lower expression of CD11a/CD18 on human neutrophils. The medium avidity state of CD11a/CD18 initiates slow rolling due to transient interactions with ICAM-1 (133, 138). This allows the neutrophil to sample chemokines presented by the endothelial cell wall, thus inducing chemokine mediated inside-out signaling, which leads to further activation of *β*2 integrins (138). Although it has been found that selectins are sufficient to cause *β*2 integrin-mediated arrest (139), both the presence of selectins and chemokine signals may be required for full activation of CD11a/CD18 and CD11b/CD18 in human blood vessels (138), as selectins fully activate *β*2 integrins cooperatively at low chemokine concentrations (104). In human neutrophils, chemokines but not selectins used PI3K*γ* in cooperation with Rap1a to mediate integrin-dependent slow rolling (at low chemokine concentrations) and arrest (at high chemokine concentrations). High levels of chemokines activate *β*2 integrins without the need of selectin signals.

### Role of *β*2 Integrin Downstream Signaling in Slow Rolling, Adhesion, Firm Adhesion, and Crawling

Upon interacting with the vascular wall, neutrophils first decrease their velocity, strengthen their interaction with the endothelium, and finally sample the endothelium by crawling over it to find an appropriate spot to squeeze through and transmigrate into the subendothelial space. In humans, both CD11a/CD18 and CD11b/CD18 have partial redundancy (131), whereas CD11b/CD18 seems redundant in terms of adhesion compared to CD11a/CD18 in mice (140).

For adhesion strengthening, *β*2 integrins can upregulate their own affinity state. Upon engagement of *β*2 integrins with their ligands, SFKs are recruited in order to phosphorylate SYK, among other proteins. In mice, the absence of HCK and FGR proteins prevents continued adhesion to ICAM-1 (141), indicating that *β*2 integrins regulate themselves to maintain a high-affinity conformation. Another example is the binding of 14-3-3*ζ* isoform to *β*2 integrins upon phosphorylation of Thr578 of the CD18 cytoplasmic tail (142), although a membrane-proximal site of the cytoplasmic tail which does not require phosphorylation has also been described (143). The binding of 14-3-3*ζ* to phospho-Thr578 obscures the binding site for Filamin-A (142), a protein which keeps *β*2 integrins in an inactive state (144). Some of the signals may counter or act to fine-tune the process of adhesion strengthening. This has been indicated in mice by the promoting activity of PKCs (145), whereas hematopoietic progenitor kinase 1 (HPK1) in concert with mammalian actin binding protein 1 (mAbp1) specifically activates CD11a/CD18 (146), thereby negatively affecting intraluminal crawling and spreading of neutrophils.

Aside from regulating their own affinity state, CD11a/CD18 and CD11b/CD18 each also separately clusters with itself. This increases the overall binding avidity of the integrins, which is required for firm adhesion. Integrin clustering also facilitates outside-in signaling by grouping the CD18 cytoplasmic tails close together. Clustering likely occurs due to the linkage of CD11a/CD18 and CD11b/CD18 to the actin cytoskeleton, in which filamentous actin (F-actin) polymerization plays a pivotal role. The initial F-actin polymerization likely is not induced by direct stimulation of CD11a/CD18 or CD11b/CD18, but rather through surface receptors for tumor necrosis factor α (TNFα) and other chemokines (147). Blocking specifically actin polymerization with Cytochalasin D, a strong actin polymerization inhibitor, prevents adhesion strengthening and crawling but does not change CD11a/CD18 or CD11b/CD18 expression in human neutrophils (148). Prior to F-actin polymerization, the SFKs HCK and FGR as well as SYK relocate to the cytoplasmic tail of CD11a/CD18 or CD11b/CD18 (149), and mouse knockout models highlight the relevance of these proteins in spreading and F-actin polymerization (101, 141, 150–152). These studies collectively indicate that F-actin polymerization occurs prior to adhesion strengthening and clustering and that *β*2 integrins relay signals for actin polymerization. After initial F-actin polymerization, integrins recruit multiple actin binding proteins, including Vinculin and *α*-actinin (43, 153, 154). Neutrophils express the cytoskeletal protein Vinculin, although they do not form mature focal adhesions as macrophages or fibroblasts do. The role of Vinculin in *β*2 integrin-dependent neutrophil adhesion, migration, and recruitment was found to be not completely abrogated in knockout mice and more so under static than dynamic flow conditions, suggesting a dispensable ‘mechanosensitive’ function *in vivo* (155). Other proteins might also promote integrin clustering. The protein 14-3-3*ζ*, which binds the phosphorylated Thr^578^ in the CD18 cytoplasmic tail, forms dimers which indicates a role in clustering of *β*2 integrins (36, 92, 156). Moreover, Cytohesin-1 increases firm adhesion by CD11a/CD18 but decreases adhesion by CD11b/CD18, indicating a role for this protein in the regulation of integrin clustering (98, 99, 127–129). The recruitment of actin binding proteins and CD18 binding proteins ultimately packs the *β*2 integrins close together due to their collective binding to F-actin filaments close to the membrane, resulting in an increase in binding avidity. It remains to be shown which of these actin-binding proteins are non-redundant in neutrophil behavior. Interesting novel candidates involved in F-actin dynamics might be found in proteomics data from MKL1-deficient patients, which show downregulation of actin-associated proteins AIP1, ARHGAP9 and Profilin-1 (157), regulating actin polymerization and disassembly (AIP1, ARHGAP9), or inhibits polymerization of actin (ARHGAP9, PFN1).

### Role of *β*2 Integrin Downstream Signaling in Cell Polarization, Spreading, Chemotaxis, and Migration

Once inside-out signaling facilitates *β*2 integrin intramolecular conformational changes, the outside-in *β*2 integrin signaling, which is subsequently initiated by adhesion, automatically takes place. Adhesion is followed by polarization of the cell for subsequent directed cell migration or chemotaxis by recognition of chemoattractants such as chemokines, lipid mediators, or chemotactic peptides (158).

The process of chemotaxis can be divided in three separate events: sensing of the chemoattractant, polarization, and cell motility or migration. Here we describe 2D chemotaxis. In fact, chemotaxis in a 3D environment is mechanistically a very different process and can occur even without integrin-mediated adhesion (159). Upon perceiving chemoattractants, leukocytes typically invoke changes in the actin cytoskeleton and activate their *β*2 integrins as discussed previously. These cytoskeletal rearrangements initiate the second step of cell polarization (160, 161) as initiated by translocation of intracellular and extracellular proteins towards the leading edge of the cell (160, 161). In contrast to tissue cells, leukocytes migrate by forming pseudopods or lamellipodia, actin-filled protrusions important for cell movement at the leading edge of the cell (162). At the trailing end, cells form a uropod which serves as the anchoring point of the cell. For motility to occur, the pseudopod needs to move towards the chemoattractant, adheres with *β*2 integrins closer to the chemoattractant, and creates a strong interaction between the actin cytoskeleton and the *β*2 integrins. Subsequently, the uropod pulls the trailing end of the cell towards the new site of adhesion, which requires these strong interactions between the actin cytoskeleton and the *β*2 integrins. Meanwhile, the uropod also degrades the F-actin at the trailing end after this movement. Concluding, the turnover of *β*2 integrin interactions at the uropod is critical for proper regulation of motility [also reviewed in (160)].

**Figure 2:**
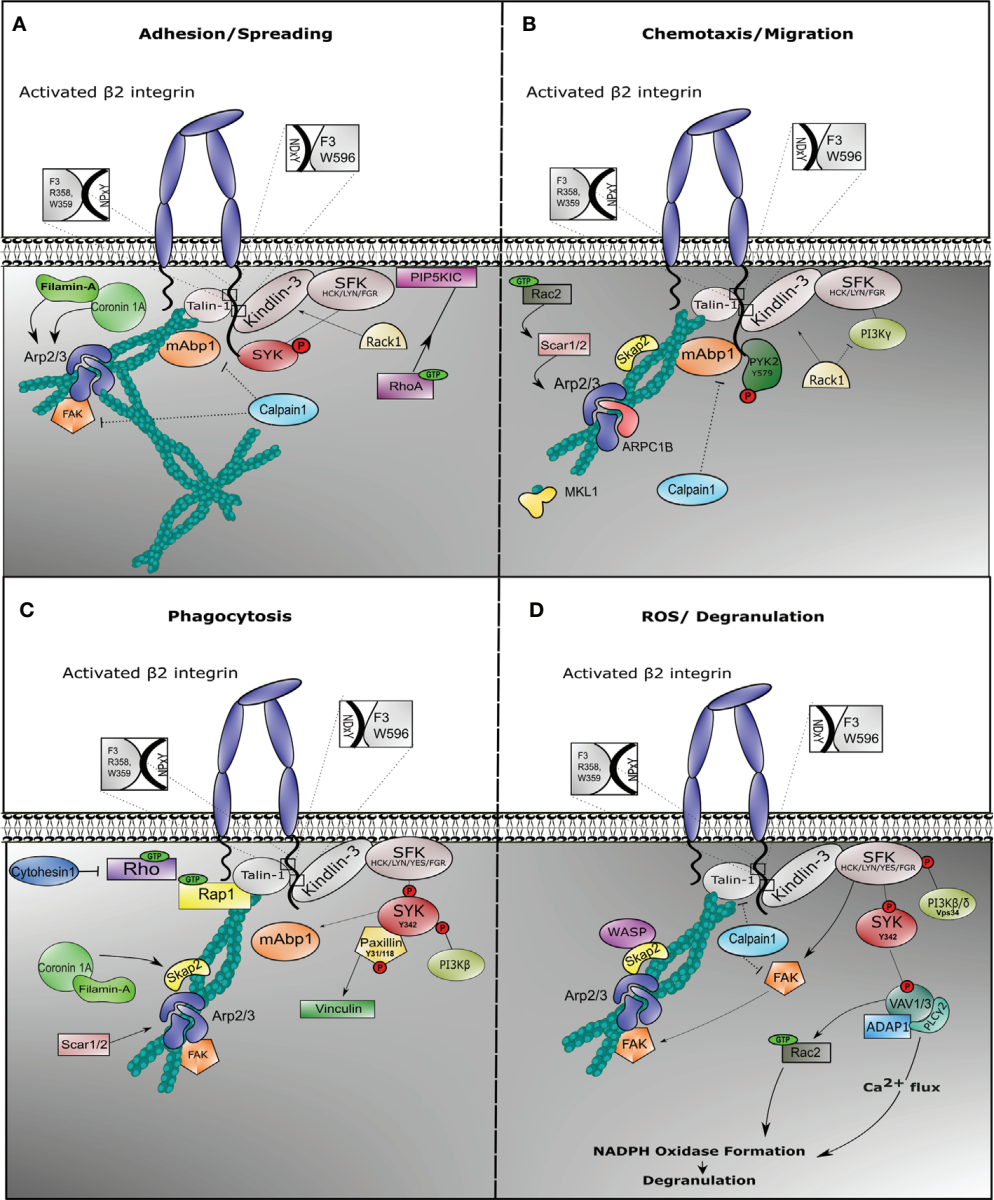
Outside-in signaling involves different protein complexes in response to neutrophil effector function. Once *β*2 integrin is activated on neutrophils, outside-in signaling is facilitated in order to induce cell effector function. Neutrophils are endowed with multiple mechanism, all achieving target elimination. In all cases, integrin outside-in signaling involves stable interaction with Talin-1 and Kindlin-3, which recruits binding of the actin cytoskeleton and induction of signaling *via* Sarcoma family kinases (SFKs). During spreading **(A)**, SFKs phosphorylate and recruit SYK to the cytoplasmic tail of the *β*2 integrin, while Phosphatidylinositol 4-phosphate 5-kinase (PIP5KIC) induces activation of RhoA GTPase. Mitogen-activated binding protein 1 (mAbp1) is involved in stabilization of actin cytoskeleton, while Calpain-1 could play a role to negatively regulate its function. During migration **(B)**, phosphorylated PYK2 is recruited and the Arp2/3 complex is stabilized *via* SKAP2 and Rac2 GTPase. Filamin A and ARPC1B are important for migration. We do not show WASP protein because deficiency results in impaired neutrophil migration in murine models only. Particularly during phagocytosis **(C)**, phosphorylation of tyrosine protein kinase SYK induces binding of phosphorylated Paxillin with subsequent activation of Vinculin and activation of Phosphoinositide 3-kinase (PI3K*β*) and mAbp1. SCAR1/2 protein (also known as WAVE1/2) recruits Arp2/3 complex to stabilize actin cytoskeleton, while SKAP2 (or SKAP55R) functions as adaptor protein. During ROS production **(D)**, degranulation is facilitated through increase of calcium influx. Phosphorylation of SYK initiates VAV phosphorylation with ADAP1 and PLC*γ*2 complex formation, promoting oxidative burst. VAV induces Rac1 GTPase activity, which facilitates NAPDH oxidase formation. In this context, Calpain1 could additionally inhibit Talin-1 or Focal adhesion kinase (FAK) function.

Following adhesion, activated integrin induces tyrosine phosphorylation of SYK protein by SFKs, such as HCK, FGR, or LYN in neutrophils (101, 163) Activation of SYK results into provisional binding to CD18 molecule, which accordingly facilitates rearrangements of the actin cytoskeleton and subsequent neutrophil spreading (**Figure 2A**). In mice, neutrophil SYK deficiency resulted in defective adhesion-dependent responses, *i.e*. spreading, degranulation, and oxidative burst. Yet, neutrophil migration was not impaired in the absence of SYK, indicative of the variation of integrin signaling in each neutrophil response (101).

During *β*2-mediated polarization of myeloid HL-60 cells differentiated towards neutrophil-like cells (dHL-60), mAbp1 was enriched towards the cell’s leading edge, suggesting that mAbp1 is a positive regulator under *β*2 integrin-mediated chemotaxis by counteracting the more adhesion-strengthening signals, thus enabling cell motility (164, 165). HPK1, a protein colocalizing with mAbp1 and actin, was required for CXCL1-induced CD11a/CD18-mediated adhesion in dHL-60 cells, independent of CD11b/CD18 proteins (146). CD11a/CD18 is known to be endo- and exocytosed in a lipid-raft mediated coordination in the presence of chemokines in contrast to CD11b/CD18 (166), again supporting distinctive roles of CD11a/CD18 compared to CD11b/CD18 in the process of cell motility.

Filamin-A was found to be indispensable for dHL-60 spreading but was not essential for neutrophil migration (167). Although Filamin-A is shown to directly interact with Coronin-1A, deficiency of the actin-binding protein Coronin-1A resulted in reduced spreading and impaired chemotaxis, which indicates an independent role of these two proteins during neutrophil motility (96). This might be explained by the role of Coronin-1A in recruiting Arp2/3 complex to the F-actin polymerizing filament (168). Arp2/3 complex is essential to coordinate actin cytoskeleton rearrangement during chemotaxis, while Filamin A may act in more static conditions such as spreading and phagocytosis, and a negative regulator of motility (see below) (**Figure 2B**).

ARPC1B functions as a subunit of the Arp2/3 complex in hematopoietic cells, and its deficiency in neutrophils resulted in significant defective migration. Additional proteins have been shown to play a role in motility. For instance, human neutrophils lacking Megakaryoblastic leukemia 1 (MKL1) protein which normally sequesters monomeric G-actin, show normal adhesion under static conditions but impaired adhesion under flow conditions and defective chemotaxis (157).

SRC kinase-associated phosphoprotein 2 (SKAP2) and WASP, two adaptor proteins which are also linked to the rearrangements of the actin cytoskeleton, led to impaired neutrophil migration when absent in mice (169). However, neutrophils from WASP-deficient patients show no migratory defect, indicating that murine and human neutrophils may function differently in the context of migration (170). Defects in other cytoskeleton associated proteins also result in impaired neutrophil migratory functions. One such protein is Rac GTPase, a small guanine‐nucleotide binding protein, involved in cytoskeleton rearrangement through signaling *via* SCAR1/2 (also known as WAVE1/2) and Arp2/3 complex (171). Rac2 deficiency in human neutrophils leads to significantly impaired integrin-dependent chemotaxis (172).

Proline-rich tyrosine kinase 2 (PYK2) has a more complex activity. It is known to interact with cytoskeleton-interacting proteins. It associates with *β*2 integrin cytoplasmic tail upon fMLP stimulation (173) and was shown to contribute to migration in all-trans retinoic acid (ATRA)-differentiated NB4 myeloid cells (174). Pyk2 deficiency in murine model showed impaired neutrophil migration, with normal phagocytosis and killing (175). PYK2 regulation may be related to differential phosphorylation of PYK2 itself. This was observed for murine CD11a/CD18 during chemotaxis and motility where decreased migration was the result of a defect in the detachment from ICAM-1 at the trailing edge when PYK2 function was inhibited (176).

Although the above mentioned proteins are mainly important to positively regulate *β*2 integrin during motility, negative regulators have also been identified with the exception of the activated C kinase 1 receptor (RACK1). RACK1, a binding partner of G*βγ* heterotrimeric subunits, inhibits PI3K*γ* activity (177), and RACK1 deficiency showed enhanced neutrophil chemotaxis (177). Calpain-1 acts as a negative regulator of neutrophil chemotaxis downstream of GPCRs. Even though inhibition of Calpain-1 promoted random migration of resting neutrophils through MAPK and PI3K activation, directed neutrophil chemotaxis was defective in the absence of Calpain-1 due to the lack of directionality (178, 179).

Filamin A has clearly been found to bind to *β*2 integrin tails at a site which overlaps with Talin and Kindlin-3 (142), and its binding is regulated by phosphorylation of the conserved threonine triplet, where Filamin A can only bind to the unphosphorylated integrin. Filamin A is a large cytoskeletal protein which interacts with a multitude of intracellular partners and has been reported to function as an integrin negative regulator by competing with Talin-1 (180). Indeed, murine neutrophils lacking Filamin A display increased integrin-mediated adhesion of T cells (97), confirming that Filamin A indeed functions as a negative regulator of *β*2 integrins in these cells. Moreover, neutrophil extravasation in a mouse peritonitis model is affected negatively by both conditional knockout of Filamin A or Talin-1; thus correct interplay between positive and negative regulators is crucial for normal neutrophil function and behavior (63, 181).

Interestingly, we have also found that SHARPIN, which binds to integrin *α*-tails, can negatively regulate *β*2 integrin-mediated neutrophil adhesion (Khan, Fagerholm, unpublished observation), indicating that integrin binding proteins indeed can directly regulate integrin function in both positive and negative ways in neutrophils.

It is undeniable that neutrophil function requires a well-balanced equilibrium of positive and negative regulation, yet further proteins that negatively regulate neutrophil chemotaxis in a integrin-dependent manner have not been established (182). Although protein kinases such as SYK and SFKs mediate PMN activation through ITAM signaling domains, SFKs also activate ITIM domains to negatively regulate PMN signal transduction pathways. ITIM phosphorylation by SRC family kinases (HCK, FGR and LYN) recruits SH2 domain-containing phosphatase (SHP) 1 and 2 as well as SHIP 1 and 2 that dephosphorylate substrates of ITAM mediated activating signaling pathways in order to downregulate PMN functional responses. These ITIM containing immunoreceptors may play key roles in negative regulation, including chemotaxis. Well known is the effect of a point mutation in *Shp-1* that reduces expression of SHP-1 protein by 90% and displays a hyper-adhesive phenotype of macrophages, contributing to neutrophil inflammation, mostly of the skin, in humans with a spontaneous mutation in the *PTPN6* gene encoding SHP-1 (183, 184).

## *β*2 Integrin Signaling in Neutrophil-Mediated Killing and Cytotoxicity

### Phagocytosis

Phagocytosis is the uptake of particles by a cell. Here, we describe the uptake of pathogens by professional phagocytes of the immune system (*i.e.* neutrophils, monocytes, macrophages, and dendritic cells). All phagocytes have the capacity to eliminate invading pathogens by engulfment and degradation (185). Pathogens are often coated with a number of soluble opsonins, which are successfully recognized by phagocytes, including neutrophils (186). Upon recognition of pathogens at the plasma membrane, protrusions encircle the pathogen and draw it into the phagocyte. After engulfing the pathogen, the so-called phagosome fuses with granules in order to degrade the engulfed pathogen. Neutrophil phagocytosis is efficiently initiated upon IgG or iC3b binding to Fc*γ* receptor or complement receptors, respectively, including CD11b/CD18 (or CR3), while no role for the CD11a/CD18 integrin is described in phagocytosis (187). Earlier studies have primarily focused on macrophage phagocytosis, where it was suggested that Fc*γ* receptor and CD11b/CD18 integrin-related induction of phagocytosis are two distinct mechanisms (188) (Freeman et al., 2016). Referring to neutrophil phagocytosis, cells from LAD-I patients failed to phagocytose C4b-opsonized sheep erythrocytes or iC3b-opsonized *S. aureus* due to the specific lack of CD11b/CD18 integrin (189). In fact, CD11b/CD18 integrins and downstream associated proteins are important in both Fc receptor and CR3-induced phagocytosis (190, 191). The extracellular binding site of CD11b/CD18 (CR3) for iC3b is different from the binding site of cellular ligands (REF). In macrophage cell lines the mechanism of phagocytosis by CD11b/CD18 involves Arp2/3 complex formation for an efficient Talin-1-dependent actin-induced internalization of the pathogen. Whether a similar role of Arp2/3 and SYK in phagocytosis applies to human neutrophils is doubted. Neutrophil phagocytosis in patients deficient in ARPC1B or MKL1 is unaffected (192). Also, Arp2/3-inhibited control neutrophils did not show a defect in phagocytosis. We believe that these Arp2/3 processes in macrophages and neutrophils differ. We know that the importance of Rap1 GTPase in phagocytosis is highlighted in murine macrophage cell lines (193, 194) as well as in murine neutrophils, in which disruption of the Rap1/Talin-1 interaction abolishes phagocytosis of serum-opsonized *E. coli* (111, 195). The complementary function of Talin-1 and Kindlin-3 is also reflected in neutrophil phagocytosis, where LAD-III-derived neutrophils were unable to phagocytize zymosan particles (196) (**Figure 2C**).

SYK protein initiates integrin inside-out activation through activation of the Rap1/Talin-1 complex, while it is also involved in *β*2 integrin-induced phagocytosis stabilizing the outside-in signaling (132, 197). Phosphorylated SYK (phospho-Tyr342) colocalizes in macrophage phagosomes with phosphorylated Paxillin (phospho-Tyr31 and phosphor-Tyr118), which leads to the recruitment of Vinculin (198) and subsequent anchorage of F-actin to the membrane to facilitate target internalization. Yet, the above mentioned phosphorylation sites, somehow anchorage points for actin rearrangements, have not yet been verified during neutrophil effector functions.

The process of adhesion strengthening, spreading, or phagocytosis indicates a role of the cytoskeleton in which *β*2 integrins must provide together with additional proteins anchorage points for membrane rearrangements. *In vitro* studies have shown localization of Filamin A at the phagocytic cup during phagocytosis (167), but at least murine neutrophil phagocytosis can occur without Filamin A (97). Nonetheless, *FLNA* mutations identified thus far in humans have not indicated a major impact, suspect of a clear impact and clinical manifestations suggesting a LAD-like phenotype (199).

In contrast, the Filamin A-binding protein Coronin-1A was present only during late phagosome formation (167) and most is likely to be redundant and dispensable (97). CD11b/CD18-dependent particle ingestion required the presence of the Arp2/3 protein complex in murine macrophages (200, 201), but human neutrophils from ARPC1B-, MKL-1- or WASP-deficient patients could efficiently phagocytize and kill pathogens *in vitro* (157, 170, 192), indicating that involvement of Arp2/3 or actin-binding proteins in neutrophil phagocytosis is redundant.

The role of focal adhesion kinase (FAK), also known as cytoplasmic protein tyrosine kinase-2 (PTK2), is interesting in this respect. FAK has been explored in knockout mice, showing impaired phagocytosis of opsonized *E. coli* (202). FAK deficiency did not result in abnormal cell migration, implicating a role for this protein in stabilization of the phagocytic cup. Human studies on FAK in neutrophils and macrophages are lacking to date. Recently the role of SKAP2 has been described during murine neutrophil phagocytosis, showing impaired phagocytosis of *E. coli* particles by SKAP2-deficient neutrophils (169). The role of additional proteins in this process still needs to be determined. In this context, signaling molecules such as SYK and PYK2 are important kinases that may contribute to the upstream integrin-mediated signaling required to initiate or stabilize in conjunction with FAK, the actin-based spreading or particle uptake in macrophages or neutrophils.

Last, in contrast to neutrophil chemotaxis, neutrophil phagocytosis is promoted in the presence of Calpain-1 (203). Human neutrophils incubated with Cytohesin-1 inhibitor SecinH3 resulted in significantly increased phagocytosis of unopsonized zymosan (204). Cytohesins are a class of small GEFs for ADP-ribosylation factors (ARFs), which regulate cytoskeletal organization, integrin activation, or integrin signaling, being ubiquitously expressed. In neutrophils, Cytohesin-1, in contrast to the above mentioned proteins, seems to be a negative regulator of phagocytosis, maintaining the equilibrium in integrin regulation.

### Antibody-Dependent Cellular Cytotoxicity

As mentioned, the presence of immunoglobulin can initiate Fc receptor activation and facilitate phagocytosis in an integrin-dependent way. When a target is too large to be phagocytized, neutrophils can exploit immunoglobulin binding to perform antibody-dependent cellular cytotoxicity (ADCC) (205). Besides antibody-opsonized bacteria and fungi, neutrophils can also kill antibody-opsonized covered targets such as cancer cells by extracellular toxicity (206, 207). Although Fc*γ* receptors have been traditionally invoked as the main receptors for antibody binding (208), also Fc*α* receptors are capable of initiating ADCC in human monocytes and neutrophils (209). Activation of these Fc receptor induces an intracellular signaling cascade with the immunoreceptor tyrosine-based activation motifs (ITAMs) of the Fc tail being phosphorylated, thus recruiting SYK protein binding, which eventually facilitates ADCC response. In ADCC of target cells, cell–cell interactions take place, gearing the response to a certain strength and outcome.

The *β*2 integrins are involved in ADCC either *via* inside-out or outside-in integrin regulation (210). CD11b/CD18 can become activated from Fc*γ* receptor IIa or IIIb through the recruitment and activation of PI3K. To date, the most solid evidence on *β*2 integrin and FcR involvement during ADCC has been collected from natural killer (NK) cells (211). Moreover, both CD11a/CD18 and CD11b/CD18 ligation to ICAM-1 positively regulates conjugate formation and cytolytic granule release (212, 213), whereas PD-1 signaling impairs outside-in integrin signaling and subsequent granule polarization (214). Yet, the killing effector function of NK cells substantially differs from neutrophils and macrophages, as it involves NK cell granule components.

We and others have clearly implicated the substantial role of CD11b/CD18 on neutrophils in ADCC against cancer cells (215, 216); however, current evidence on the integrin-associated ligands on tumor cells remains very limited. Although *in vitro* studies have shown that neutrophils adhere to melanoma cells *via β*2 integrin–ICAM-1 interactions (217), our recent findings have excluded ICAM-1, -2 or -3 as essential ligands for *β*2 integrin during neutrophil adhesion and cytotoxic activity in the case of breast cancer cells (218), indicating that the neutrophil interactions with different cell targets may vary in the use of counter/receptors or ligands.

### ROS Production

Simultaneously with the process of phagosome formation, neutrophils consume an extreme amount of oxygen (respiratory burst), which enables them to produce reactive oxygen species (ROS) (219). The enzymatic machinery responsible for this function is the NADPH oxidase enzyme complex, which converts oxygen to superoxide and consequently hydrogen peroxide (220). As we have previously demonstrated, LAD-III syndrome causes impaired respiratory burst in response to zymosan, indicating a clear correlation between integrin activation and ROS production (196). These data are complementary with CD11b/CD18 deficient cells in mice, which resulted in defective respiratory burst (221). Integrin-mediated ROS induction (169) and Fc*γ*-receptor-mediated neutrophil activation required members of the VAV guanine nucleotide exchange factor and Rac small GTPase families (VAV1/3 and Rac2, respectively) (222, 223), and the PI3-kinase isoforms PI3K*β* and PI3K*δ* instead of PI3K*γ*, with a predominant role for PI3K*β* (224–226). SLP76/ADAP1 together with VAV isoforms (VAV1 and VAV3 in neutrophils) forms a complex with PLC*γ*2 which is also associated with immune complex-mediated signaling (222, 227, 228). VAV protein also functions as a Rac-GEF, enabling activation of Rac2 GTPase and thus inducing NADPH oxidase formation (229).

In an effort to further dissect the integrin-induced signaling cascade leading to NADPH oxidase in murine model systems, apart from the previously reported cascades, an indispensable role of the class III PI3K isoform (Vps34) was established in integrin-related ROS production in response to *Escherichia coli* or *Staphylococcus aureus* in neutrophils (**Figure 2D**). Vps34 is different from the above-mentioned class I PI3K isoforms (**Figure 2D**), and it is used for successful phagosome maturation and *Mycobacterium tuberculosis* destruction, while not essential for neutrophil chemotaxis or adhesion (230).

Outside-in signaling is important in adhesion-dependent responses so that neutrophils in suspension will not be able to perform, including degranulation and ROS production in case of TNF-induced effector function (231). Integrin outside-in signaling has been associated with neutrophil effector functions, including adhesion-dependent ROS production, which was dependent on SYK (101). Data on murine or human SFK-deficient neutrophils (lacking all three neutrophil-expressed SFK members, LYN, HCK and FGR) demonstrated the crucial role of SFKs during TNF-α-induced ROS production, accompanied by reduction of VAV protein phosphorylation in mice (232), which is a known regulator of oxidative burst (222).

## Therapeutic Approaches

With the emerging role of neutrophils in health and disease and because *β*2 integrins play such an integral role in the cellular functions of this cell type as discussed above, targeting *β*2 integrins on neutrophils specifically with small molecules could prove fruitful as a therapy. There are two considerations to use integrin-modulating drugs in the context of neutrophil function. Neutrophil activity could be dampened or enhanced through blockade or induction of integrin activity, respectively. For example, decreasing neutrophil extravasation could provide major benefits to patients suffering from inflammatory bowel disease such as Crohn’s disease (233, 234), whereas LAD-III patients would benefit from an increase in neutrophil extravasation (80, 196, 235). One absolute requirement for such therapy in neutrophils would be the high specificity for the *β*2 integrin molecule due to the multifunctional nature of *β*2 integrins, as well as of other proteins involved in its downstream signaling cascade. The way of intervening neutrophil function by altering *β*2 integrin activity could vary. A large selection of small molecules has been shown to interact with CD11b/CD18, giving rise to various candidates to be further explored as therapeutics (236), and low dosage of such compounds might also already impose a sufficient response with limited side effects on neutrophils and other leukocytes.

Another interesting possibility is the use of antibody-based therapies. A recent study has shown the use of a monoclonal antibody, named anti-M7 that specifically disrupted the interaction between CD11b/CD18 and CD40L and not any other tested ligand. In blocking this CD40L-binding activity of CD11b/CD18, downregulation of pathological inflammation in mice could be accomplished while keeping other functions intact (237). In contrast to general anti-CD11b antibodies (238), this anti-M7 against the ligand-binding I-domain of the CD11b subunit ameliorated instead of aggravated sepsis. Anti-M7 in fact enhanced iC3b binding and phagocytosis while blocking binding to endothelial or platelet CD40L (237). Such ligand-specific antibodies might prove useful during acute inflammatory responses or sepsis. One possible downside to the usage of antibodies is their relatively long half-life and possible off-target effects on other leukocytes, but this might be altered by tempering with the antibody structure, isotype, or using nanobodies which have a much shorter half-life. Humanized monoclonal antibody against CD11a (Efalizumab) has been thoroughly studied in a Phase III clinical trial in psoriasis patients, but the drug has been withdrawn due to a significant risk of developing multifocal leukoencephalopathy (239, 240). Collectively, intervening with *β*2 integrin-mediated interactions seem to be a promising avenue in both decreasing neutrophil extravasation as well as trying to increase neutrophil phagocytic activity, albeit requiring very specific targeting, dosage, or both, which seems a very unique characteristic to explore in case of the CD40L binding sequence in the I-domain of CD11b (237).

Another possibility in regulating neutrophil integrin function is by modulating other proteins involved in either inside-out or outside-in signaling. This would allow for intervention in integrin activation and thus function, but also modulation of specific processes, such as phagocytosis or ROS production, while excluding or limiting the effects on other cellular processes. For inside-out signaling, Kindlin-3 might be a promising candidate. However, this would require small cell-permeable Kindlin-3-antagonists which specifically interfere with *β*2 integrin activation while preserving activation of other integrins. This effect might be achieved by obscuring binding between the threonine triplet in the CD18 cytoplasmic tail, since threonine into alanine mutations affect the Kindlin-3 interaction with CD18 in T cells while retaining partial activity (241). Notably, this motive is absent next to the second NXXY motif of other *β* integrins and could be a specific target for *β*2 integrins (242), thus avoiding bleeding, a side effect also seen in LAD-III patients. Recent work has also elucidated for the first time the crystal structure of Kindlin-3 (67), which might partially reside as an auto-inhibitory homotrimer. Relaxation or enforcement of this homotrimeric state might be another way in which inside-out signaling can be influenced either positively or negatively, respectively.

Aside from Kindlin-3, Cytohesin-1 is an interesting molecule as a drug target since its inhibition results into neutrophil pre-activation, although its function is also implicated in phagocytosis (204). Since this effect might stem from the relief of negative regulation by Cytohesin-1 on the Arf6 GTPase (98), exploring specifically Arf6 inhibitors or activators could also prove to be a valid therapy as well as expand our current knowledge on its role in integrin activation. Although targeting inside-out signaling shows potential, targeting outside-in signaling proteins solely involved in one cellular process could also be of interest to retain other cellular functions associated with *β*2 integrins. For example, targeting PI3K*δ* could be sufficient to downregulate ROS production and degranulation, although having a minor role compared to PI3K*β* (224–226). Moreover, Pyk2 could be a good target to reduce chemotaxis and migration while keeping other functions intact (175, 176). In conclusion, intracellular proteins regulating integrin activity could be explored as potent therapeutic targets; however, taking into account the multifunctional roles of the proteins in neutrophil response, more studies should be performed to assure maximal efficacy with limited off-target effects.

As mentioned above, Calpain-1 is a negative regulator of integrin-dependent chemotaxis and migration by cleaving a number of integrin-associated proteins such as Talin-1 (243). Calpains are intracellular cysteine proteases with very diverse physiological roles. *In vitro* studies with human neutrophils and small inhibitory compounds have shown that Calpain-1 is also involved in the *β*2 integrin signaling cascade of adhesion-dependent TNFα-induced oxidative burst (244). The potential use of therapeutic Calpain-1 inhibitors to counter predictable and unwanted inflammatory reactivity in which neutrophils play a major role, has been explored (244). Yet, aberrant activity of calpains—whether it be over- or under-activation—is often associated with pathological functions and thus with diseases. Effective calpain inhibition may be achieved by peptide and peptidomimetic inhibitors developed to increase the specificity and potency. Since calpains constitute a family of homologous proteins ubiquitously expressed, highly specific inhibition seems problematic. Calpain inhibitors have demonstrated efficacy in animal models of calpain-related diseases, but progression of the inhibitors into clinical trials has been hampered partly due to the lack of calpain isoform selectivity of these inhibitors.

Worth mentioning is the alternative approach for targeting *β*2 integrins with ICAM-1 blocking antibodies. Studies in patients with partial-thickness burn injury using the anti-ICAM-1 antibody (Enlimomab) have shown promising modulation of the inflammatory response and improved wound healing (245). However, application of the drug has also shown several side effects. Additional *ex vivo* studies have shown that the concentration of the antibody used in the initial clinical trials could promote complement-dependent neutrophil activation (246). For the treatment of Crohn’s disease or ulcerative colitis, a 20-base antisense oligonucleotide inhibiting ICAM-1 production (Alicarfosen) showed potential clinical benefit (247, 248). Clinical remission rates compared to placebo and whether the response can be maintained in the long-term in larger studies are as yet unknown.

## Discussion

We have reviewed and discussed most neutrophil functions requiring signaling through *β*2 integrins, either inside-out or outside-in integrin-dependent neutrophil activation. Although several proteins downstream of *β*2 integrins have been identified to play a role in phagocytosis, degranulation, or respiratory burst (**Table 1**), their exact role and sequential order or interdependence in integrin-mediated outside-in signaling cascade in killing and cytotoxicity remain to be determined. However, additional studies with innovative technologies are needed to develop a full picture of the CD11b/CD18 or CD11a/CD18 signaling cascades and their involvement in neutrophil effector function in humans.

**Table 1:** Relevance of human and mouse CD11a/CD18 and CD11b/CD18 in neutrophil function.

	*Human CD11a/CD18*	*Human CD11b/CD18*	*Mouse CD11a/CD18*	*Mouse CD11b/CD18*
***Adhesion/Crawling***	Initial adhesion of intermediate-avidity to endothelium following selectin-mediated rolling. After firm CD11b/CD18-mediated adhesion, a role in crawling over the endothelium (131)	Suggested to become fully active after CD11a/CD18. Pivotal in firm adhesion to the endothelial cell wall (131)	Most pivotal of the CD11/CD18 isoforms in mice, KO results in severe adhesion defects despite presence of CD11b/CD18 (249)	More redundant in mice, but adhesion and migration is affected in its absence (249).
***Antibody derived cellular cytotoxicity (ADCC)***	N/A	Activated *via* Fc*γ*-receptors, involved in downstream signaling (216).	N/A	N/A
***Chemotaxis/spreading (Two-dimensional)***	Turnover of CD11a/CD18 during chemotaxis (166).	Increased avidity of CD11b/CD18 at cell-contact sites (250).	CD11a/CD18 seems more important compared to CD11b/CD18 in mice (176).	N/A
***Phagocytosis/ROS production***	No role described for CD11a/CD18 (251).	Binding of pathogens in a lectin-like way or once opsonized with iC3b and C4b (189). Recruits Fc*γ*-receptors for downstream signaling (196).	N/A	Binding of complement-opsonized zymosan. Recruits Fc*γ*-receptors for downstream signaling (221).

Summary list of human or murine studies regarding β2 integrin activation, inside-out or outside-in signaling during neutrophil effector function.

The last decade, the technology of unbiased OMICS has improved our knowledge in cell biology. From transcriptomics to proteomics, we are now able to characterize RNA and protein expression levels in primary cells with an example of dissecting the composition of neutrophil cytoskeleton and the proteins associated with phagosome maturation (252, 253). In addition, large-scale studies include identification of signaling events and phosphorylation patterns after neutrophil stimulation, all of which have been nicely summarized elsewhere (254). Similar approaches have been followed in case of *β*1 adhesion receptors or intracellular signaling, where integrin-associated complexes have been identified by proteomic analysis (255–257). Recent methods focusing on interactome mapping based on proximity, such as Enhanced Ascorbate PeroXidase (APEX) or Biotin IDentification (BioID) have been used in order to anticipate function or downstream signaling cascade of *β*1, *β*4, and *β*5 integrins (258–262). Yet the complexity of leukocyte- associated *β*2 integrin has not allowed the investigation of its pathway with such method to date.

The increasing knowledge on the *β*2 interactome and integrin signaling in the context of neutrophil function allows it to be considered as a target for therapeutic intervention. Due to the short half-life of primary human neutrophils, research on *β*2 integrin-associated proteins upon activation is very limited. An alternative way to explore *β*2 integrin signaling is by exploiting neutrophil-like cell lines, such as HL-60 and NB4 cells. However, a considerable disadvantage of such cell lines is that they do not completely recapitulate neutrophil morphology or function, still unable to produce granules or migrate. Studies with these myeloid cell lines are useful for an initial orientation but will not be able to reveal the precise mechanism of signaling events in primary cells (263). Therefore, current efforts are focusing towards the generation of reproducible ways to generate neutrophils derived from induced pluripotent stem cells (iPSCs), being much closer to primary neutrophils (264).

In summary, we have discussed the role and signaling events of *β*2 integrins during neutrophil effector function. The relevance of this protein in this context is undisputed; however several questions remain unanswered. Although a detailed common pathway for extension of the *β*2 integrin is known, such a pathway for high-affinity activation of *β*2 integrins remains elusive; therefore we have summarized the current and most recent findings. We have also described differences regarding proteins involved with *β*2 integrins during different neutrophil functions such as adhesion, oxidative burst, phagocytosis or ADCC, highlighting the notion that *β*2 integrin outside-in signaling might include several signaling pathways, each distinctive per neutrophil effector response. Due to short neutrophil half-life, most studies have been focused on murine neutrophils, yet the differences compared to human neutrophils are substantial. To avoid possible confusion and emphasize the potential impact of each study, we have highlighted the data derived from mouse and human neutrophils. To conclude, usage of novel methods for both analysis and generation of neutrophils will be a key to further understanding the *β*2 integrin signaling. Such studies could lead to new therapeutic targets for modulating *β*2 integrin mediated neutrophil functions, or even leukocyte function in general.

## Author Contributions

HM and TK were the principle investigators that designed the work. SF, RA, MM, HM, and TK supervised the work on behalf of the LADOMICs consortium. PB, SW and MM wrote the manuscript. All authors contributed to the article and approved the submitted version.

## Funding

This work was supported by the Dutch Cancer Society [grant number KWF 11537] and eRARE [eRARE, LADOMICs JCT2018/ZonMW 90030376506], Academy of Finland, E-RARE (Academy of Finland), University of Helsinki (HiLife HIPOC), Swedish Cultural Foundation and Liv och Hälsa foundation and a grant of CIDA (Center of ImmunoDeficiencies Amsterdam).

## Conflict of Interest

The authors declare that the research was conducted in the absence of any commercial or financial relationships that could be construed as a potential conflict of interest.

## Abbreviations

ADCC, Antibody-dependent cellular cytotoxicity; ADCP, Antibody-dependent cellular phagocytosis; EGF, Epidermal growth factor; ECM, Extracellular matrix; F-actin, Filamentous actin; FAK, Focal adhesion kinase; GPCRs, G-protein coupled receptors; HPK1, Hematopoietic progenitor kinase 1; dHL-60, HL-60 cell line differentiated towards neutrophil-like cells; ITAMs, Immunoreceptor tyrosine-based activation motifs; LAD, Leukocyte adhesion deficiency syndrome; fMLP, N-formylmethionine-leucyl-phenylalanine; NK cells, Natural killer cells; NETs, Neutrophil extracellular traps; PSI, Plexin–semaphorin–integrin; PI3K, Phosphoinositide 3-kinase; PMN, Polymorphonuclear; PKC, Protein kinase C; RACK-1, Receptor For Activated C Kinase 1; ROS, Reactive oxygen species; SYK, Spleen tyrosine kinase; TNFα, Tumor necrosis factor α; WASP, Wiskott–Aldrich syndrome protein.
